# Verifying interruption of transmission or elimination of *Schistosoma japonicum* must consider schistosome infections in wild rodents

**DOI:** 10.1371/journal.pntd.0012996

**Published:** 2025-04-09

**Authors:** Qing Xu, Han-Xiang Zhang, Yu-Xin Qi, Joanne P. Webster, Da-Bing Lu

**Affiliations:** 1 Department of Epidemiology and Statistics, School of Public Health, Jiangsu Key Laboratory of Preventive and Translational Medicine for Geriatric Diseases, MOE Key Laboratory of Geriatric Diseases and Immunology, Soochow University, Suzhou, China; 2 Department of Pathology and Population Sciences, Royal Veterinary College, University of London, London, United Kingdom; University of Massachusetts Amherst, UNITED STATES OF AMERICA

## Abstract

Following the exciting announcement that Anhui Province, one of the historically most heavily *Schistosoma japonicum* endemic regions in China, has been officially declared to have achieved the goal of province-wide transmission interruption of schistosomiasis, we raise two concerns that merit further consideration. Firstly, *S. japonicum* is zoonotic, and in China it is known that humans, livestock, and wild animals such as rodents are major reservoirs of infection. While *S. japonicum* infections both in humans and bovines have recently been reduced to zero prevalence, infection persists at moderate to high levels within rodents with, for example, prevalence’s reported of up to 12.81% within Anhui Province over last 5 years. Therefore, surveillance of schistosome infections in wild animals, at least in rodents, must be included in the criteria of verifying transmission interruption or elimination in China. Secondly, regarding how the official procedure of verification of transmission of interruption was assessed, we propose that a more accurate survey design and foci selection would be based, at least in part, upon areas where schistosome infections in either humans, domestic/wild animals and/or snails have recently been documented. This is particularly urgent, not only in terms of the long-term sustainability of disease control in China, but because many other countries are now fully acknowledging the zoonotic potential of schistosomiasis and are/or will soon enter this final elimination phase as officially outlined in the WHO’s 2021–2030 Neglected Tropical Diseases Roadmap.

## Introduction

On Oct. 21, 2023, Anhui Province, one of the historically most heavily *Schistosoma japonicum* endemic regions in China [1], has been officially declared to have achieved the goal of province-wide transmission interruption of schistosomiasis [2], according to the Chinese national standard for Schistosomiasis Control and Elimination (see details in Table 1). The news is laudable and will inspire all health workers and policymakers that China’s national 2030 goal of country-wide elimination is achievable, currently defined as where no schistosome infections have been detected in humans, livestock, and snails for 5 consecutive years after transmission interruption.

**Table 1:** Chinese national standard for schistosomiasis transmission control, interruption and elimination (GB 15976-2015).

Criteria of transmission control
<1% prevalence in autochthonous humans.
<1% prevalence in autochthonous livestock.
No autochthonous acute schistosomiasis.
No infected snails for >2 consecutive years.
Criteria of transmission interruption
No autochthonous human infections for 5 consecutive years.
No autochthonous livestock infections for 5 consecutive years.
No infected snails for >5 consecutive years.
An effective county-level monitoring system.
Criteria of elimination
No infections in autochthonous humans, livestock, and snails for 5 consecutive years after transmission interruption.

However, while not to detract from the successes achieved to date, we propose here two concerns that merit further consideration, with one regarding the criteria of the national standard and the other being the protocol used for the technical evaluation.

## Criteria of the national standard need modified

**Fig 1:**
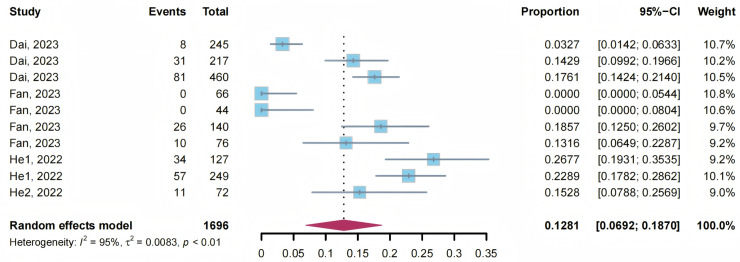
Prevalence of *Schistosoma japonicum* infections in wild rodents reported recently in Anhui of China. Horizontal lines indicate 95% CIs; horizontal points of the red diamond are the limits of the overall 95% CIs; and the dashed line shows the position of the overall prevalence. The included studies are ordered by year of publication.

*S. japonicum* is zoonotic, and while in China infection in humans and livestock are incorporated into the national standard (Table 1), it is known that wild animals such as rodents are also major reservoirs of infection [3]. China has made significant achievements in schistosomiasis control, especially in humans and bovines, where results from 2023’s national schistosomiasis surveillance found a mean of zero prevalence of *S. japonicum* infections both in humans and bovines [4]. However, schistosomiasis surveillance and control among its sylvatic hosts has long been ignored from national guidelines. This may be most pertinent when it comes to *S. japonicum* infections in wild rodents. Schistosomiasis japonica infections in rodents have been frequently reported in the research literature and often at very high prevalence levels. Furthermore, our previous meta-analyses found the infection prevalence of *S. japonicum* in wild rodents in hilly and mountainous regions (as reported between 2004 and 2018) presented an upward trend over time [5]. Indeed, a subsequent complete literature search here retrieved four additional recent research reports of wild rodent infections in Anhui province [6–9] (Table 2). Infection prevalence levels reached over 26.77% in some counties, with a pooled mean overall infection prevalence level of 12.81% (95% CI: 6.92–18.70) (Table 2; Fig 1), up from an overall 3.86% (95% CI: 2.16%–5.93%) calculated from the studies prior to 2018 [5]. Thus, the current levels of schistosomiasis in rodents in these regions are notably higher than the defining level of schistosome transmission interruption, or even than that of transmission control, as required for humans and livestock (Table 1).

**Table 2:** Recent publications with new studies on schistosome infection in wild rodents in Anhui of China after the published work [5].

Author and year of publication	Year of study performed	Town/County (or City)	Surveyed areas	No. examined	No. infected^†^	%
Dai (2023) [6]	2020	Guichi, Chizhou	15 villages	245	8	3.27
	2021	Guichi, Chizhou	16 villages	217	31	14.29
	2022	Guichi, Chizhou	15 villages	460	81	17.61
Fan (2023) [7]	2021.9–2022.11	Huashan district, Maansan	3 communities	66	0	0.00
	2021.9–2022.11	Yushan district, Maansan	3 communities	44	0	0.00
	2021.9–2022.11	Huyang town, Dangtu	4 communities	140	26	18.57
	2021.9–2022.11	Huangchi town, Dangtu	5 communities	76	10	13.16
He (2022) [8]	2018.6	Shitai county	3 villages	127	34	26.77
	2018.10	Shitai county	3 villages	249	57	22.89
He (2022) [9]	2019.10	Shitai county	3 villages*	72	11	15.28

† Note: Infections in captured rodents were determined here by identifying *Schistosoma japonicum* eggs in feces using Kato-Katz microscopy and/or miracidial hatching techniques, combined with dissection following humane euthanasia to detect adult worms and miracidial hatching from liver sections [10]; the main infected rodent species were *Apodemus agrarius*, *Rattus losea*, and *Rattus norvegicus.*

*Rodent “pest-control” measures had been implemented in two out of three villages in the year preceding the 2019 epidemiological surveys.

Such findings should surely prompt the need for the modification and upgrading of the existing national standards, where schistosome infections in wild animals, at least in rodents (and/or region-specific wild animal species [11]), must be included in the criteria of verifying transmission interruption or elimination in China. Rodents are characterized by their “rapid” life histories, with typical traits of early maturation and short gestation times, which enable them to become the most abundant mammals in the nature. Due to their dominance as commensal species (defined as “sharing man’s table”) in human-driven environments, rodents have been and/or will be the main reservoirs of a large number of known or “emerging” novel zoonotic pathogens [12,13]. Indeed, we have previously demonstrated convincing parasitological (eggs per gram within rodent stool as identified by miracidial hatching and egg counts) [14,15], behavioral (a chronobiological shift towards crepuscular/nocturnal cercarial shedding from *Oncomelania* snails in hilly regions where rodent transmission predominates) [16], and mathematical modeling (quantification of individual *R*_o_ values by definitive host species and habitat type) [3] evidence that wild rodents are now the key hosts, rather than bovines, maintaining *S. japonicum* transmission in the hilly regions of Anhui province in China. Furthermore, molecular genotyping and phylogenetic of these parasites revealed very little or no *S. japonicum* genetic differentiation among rodent, (dog) and human definitive host species, suggesting frequent *S. japonicum* gene flow, and thus also transmission, across these species [17]. Likewise, multiple recent field surveys performed in Anhui province also identified *S. japonicum*-infected snails where rodents serve as potential reservoirs [18]. Therefore, there could be no true transmission interruption nor elimination at the province level in Anhui declared if considering infections in wild rodents.

## Procedure for evaluation of transmission of interruption needs modified

**Fig 2:**
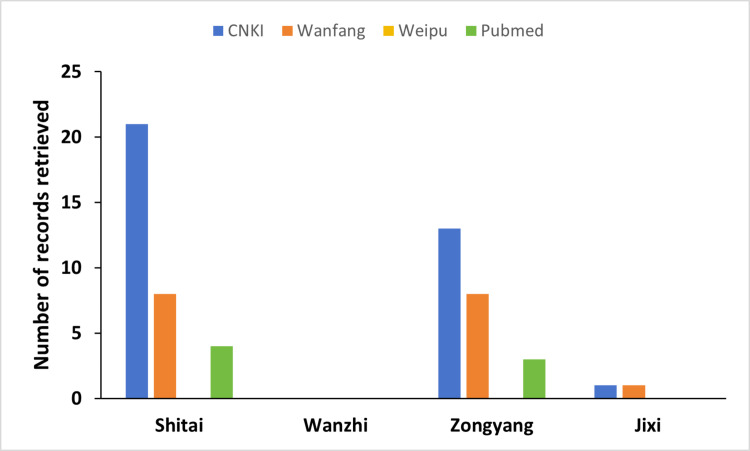
Number of records on *Schistosoma japonicum* in four regions retrieved from four databases (i.e., three Chinese CNKI, Wanfang and Weipu, and one English Pubmed) published from Jan 1, 2010 to May 28, 2024. Search terms “xuexichong” AND each of “Shitai, Wanzhi, Zongyang, or Jixi” (in pinyin) were used in the Chinese database, and search terms “*Schistosoma japonicum*” AND each of four region names in Pubmed.

We also raise potential caveats regarding how the procedure of verification of transmission of interruption was assessed for Anhui province. As reported, based on the principle of stratified random sampling, three counties/districts within Anhui providence were selected for the technical evaluation of transmission interruption standards [2]. These were Wanzhi district in Wuhu City, Zongyang County in Tongling City, and Jixi County in Xuancheng City, but excluding Shitai County in Chizhou City. However, as the main purpose was to verify if there is any infection remaining across the province, one could propose that a more accurate survey design and foci selection would be based, at least in part, upon areas where schistosome infections in humans, domestic and/or wild animals, or snails, have most recently been reported within the published and/or gray literature. Indeed, we thereby performed such a literature search across four major databases, encompassing all reports of schistosomiasis transmission documented since 2010, and found that Shitai county, the one region excluded from the verification surveillance, resulted in more positive identifications than all the three sampled areas combined (i.e., 33 versus 26; see Fig 2).

## Beyond China

The points raised here have broader implications beyond China and even *S. japonicum* transmission across Asia. Schistosomiasis remains endemic in 78 countries, with more than 90% of people requiring treatment living in Africa, where *S. mansoni* and *S. haematobium* predominate as the major forms of human intestinal and urogenital schistosomiases, respectively [19]. The revised WHO Schistosomiasis Guideline [20], Neglected Tropical Diseases Roadmap [21] and its One Health companion document [22], all propose similar 2030 elimination targets (albeit with slightly different terminology). However, very much like the Chinese targets (Table 1), while the role of livestock as potential schistosomiasis reservoirs is now acknowledged, clearer emphasis on the role of wildlife and rodents in particular needs to be incorporated, particularly when reaching the ‘verification of interruption of transmission/elimination phases’. The situation outside of Asia is more complicated for surveillance, given that multiple species of *Schistosoma* are circulating, including, for example, full acknowledgment of both *S. mansoni* and *S. haematobium* also in non-human primates, *S. rodhaini* in rodents, and *S. bovis*, *S. curassoni* and *S. mattheii* in livestock, as well as notable inter-specific hybrids (e.g., [23]). Furthermore, our recent studies have indeed not only demonstrated the existence, often at high prevalence levels, of each of these species within different species of wild rodents within Africa (e.g., through cox-1 and ITS to species level [24]), but further phylogenetic analyses (e.g., as based on the 12S rRNA gene and four protein-coding mtDNA genes: i.e., cox1, cox3, nad4, and nad3), have demonstrated shared *Schistosoma* gene-flow between humans, rodents and snails across sympatric populations [25]. While such relatively simple molecular tools alone may not necessarily indicate directionality of transmission, nor differentiate between spillover infections and key reservoir host species [26], they do provide critical surveillance markers illustrating the potential for schistosomiasis transmission persistence and re-emergence within human populations, and hence major challenges for the 2030 elimination targets.

## Action points

**Fig 3:**
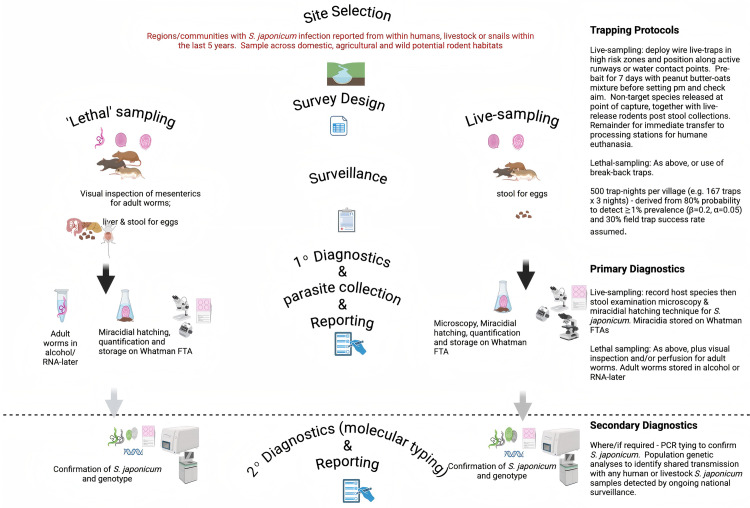
1. A proposed stratified surveillance strategy for rodent-borne *Schistosoma japonicum* transmission within mainland China (created in Biorender.com).

Given the critical importance of such sylvatic host transmission for reaching the global schistosomiasis elimination targets by 2030, we propose:

1. broader surveillance and control systems must be implemented whenever and wherever the (as using the Chinese national standard definitions, Table 1) transmission interruption targets are being reached and before any verification of elimination is declared. These should include:
  a. continued and expanded monitoring of the *Oncomelania* populations (including xenomonitoring, and following ongoing developments in environmental DNA (eDNA) surveillance [27])—and, where infections are detected (only, in order to minimize impact on the local ecological fauna and flora), implement mollusciciding;
  b. surveillance within all communities and foci wherever schistosome infections in humans, domestic and/or wild animals, or snails have most recently been reported (within the last 5 years) within the national survey reports, published and/or gray literature must be included within verification protocols; and
  c. as relates to rodent-borne (and indeed other potential sylvatic wildlife-borne) transmission surveillance, we propose a stratified surveillance strategy (Fig 3). Wherever financial and logistical constraints allow, live trapping of target rodent species (with release at point-of-capture of non-target animals and any rodents where only stool is to be collected), followed by humane euthanasia (or lethal break-back rodent trapping where necessary). Identification of *S. japonicum* infection status should be performed by the miracidial hatching technique (and, although less sensitive, microscopy) from stool and/or liver [10]. Adult worms should be collected by mesenteric inspection and/or perfusion. Both miracidial samples on Whatman FTA cards and adult worm in alcohol or RNA-later should also be stored for subsequent cataloging and genotyping. As *S. japonicum* is the only schistosome of public health currently circulating within China, secondary molecular diagnostics to species level [28] is less critical (relative to across sub-Saharan Africa for example). However, biobanking of the *S. japoncium* miracidial and adult worm samples from rodent would permit subsequent population genetic analyses enabling identification of shared human or animal host *S. japonicum* transmission [17] in situations wherever ongoing national human and bovine surveys detect new cases.
2. In terms of preventative strategies to be adopted to minimize the involvement of rodents in transmission, we are again faced with the challenge that rodents, both rats and mice, are among the most neophobic mammals known and are notoriously difficult to control, worldwide, through standard trapping and poisoning regimes. Thus, advice and sanitation strategies (WASH: water, sanitation and hygiene) to minimize the risk of rodents in close contact with humans and freshwater must be prioritized.

## Conclusions

To conclude, China has taken great efforts, and achieved huge success with, schistosomiasis control for nearly seven decades, and has accumulated rich experience. However, due to the zoonotic infection of *S. japonicum*, we firstly propose it is thereby essential to include surveillance of schistosome infections in their sylvatic hosts, notably that of wild rodents, into the current assessment mechanisms for elimination in China and beyond. Secondly, in terms of the procedure of verification of transmission of interruption, we propose a more accurate survey design and foci selection would be based, at least in part, upon areas where schistosome infections in either humans, domestic/wild animals, and/or snails have recently been documented. This is particularly urgent, not only in terms of the long-term sustainability of disease control and elimination in China, but because many other countries are now acknowledged to be endemic with potential zoonotic schistosomes [23,29], and have been and/or will soon enter this final stage as officially outlined in the WHO’s 2021–2030 roadmap [19].
